# The golden ratio—dispelling the myth

**DOI:** 10.1186/s40902-024-00411-2

**Published:** 2024-01-17

**Authors:** Farhad B. Naini

**Affiliations:** Kingston Hospital and Queen Mary’s Hospital, King’s College Hospital NHS Foundation Trust, Galsworthy Road, London, KT2 7QB UK

**Keywords:** Divine proportion, Golden number, Golden proportion, Golden ratio, Golden section

## Abstract

**Background:**

The purpose of this article is to explore the claims often cited in scientific journals regarding the golden ratio, and its proposed link to beauty and idealized forms in nature, including idealized human proportions.

**Main body:**

Claims made in the nineteenth century through to the modern day in the clinical literature do not appear to be supported by evidence.

**Short conclusions:**

There is no convincing evidence that the golden ratio is linked to idealized human proportions or facial beauty. There is currently no evidence to support the use of the golden ratio in orthognathic or facial aesthetic/reconstructive surgical planning or analysis of results.

## Background

The importance of mathematics and geometry in understanding the universe and the laws that govern it cannot be underestimated. Mathematicians and astrophysicists often talk of the beauty of a theorem or the symmetry of an equation. This link between the laws of nature and mathematics was perhaps best explained by Galileo Galilei in his *Il Saggiatore* (1623) [1, 2]:

[The cosmos/laws of nature] is continually open before our eyes, but it cannot be comprehended until we understand the characters in which it is written, and that is in the language of mathematics. Its characters are triangles, circles and other geometrical figures, without which it is humanly impossible to understand a single word. Without these, we are wandering in vain through a dark labyrinth.

With this in mind, it is perhaps not surprising that some authorities have attempted to find potential links between the morphology of objects and their perceived beauty. Moreover, there are also those who claim to have discovered the mathematical “secret” to beauty, and to convince others of their finding. The most common assertion in relation to beauty is that is it inextricably linked to an almost magical number, called the golden number, and the ratio derived from this number, the golden ratio.

Perhaps the most commonly quoted and referenced paper supporting this concept was written by the influential and pioneering orthodontist Dr. Robert Ricketts in May of 1982 [3]. A few months later he published a very similar article in the plastic surgery literature [4]. The claims made supporting the golden ratio as the underlying element in beauty were not new, but they were new to the scientific literature. The following are quotes from the former article:

“For appreciation of beauty, it has been suggested that the human mind functions at the limbic level in attraction to proportions in harmony with the Golden Section.

The normal human face is possibly the most beautifully perfect structure in all of the animal kingdom.

This golden sectioning seems to have some marvelously unique properties. It is a quality which, for some reason, attracts the attention and is recorded in the limbic system as beauty, harmony, and balance.

There is a certain quality of the golden section which stimulates the viewer,

Because a famous Greek sculptor, Phidias, used the golden proportion so much, it was called phi.

…the golden rectangle. It was on such a scale that the Parthenon was built, and it has endured for two millenniums as a world attraction.

With the foregoing biologic facts in mind, it was only natural to examine faces for alternative phi relationships.

In the final analysis, in the frontal view a natural progression takes place in the face (if it is beautiful)!

It would appear that the principle of the golden section and Fibonacci numbers are basic to this orderly arrangement and growth of the human face.

… the thought was pondered that perhaps, just perhaps, basic mitosis and cell division are also monitored by factors in Fibonacci numbers!

Those of religious persuasion may take comfort from this magnificent and majestic organization of the human face.”

Over a decade and a half later, Dr. Ricketts continued his support for this concept, writing: “Underlying many treatment decisions is the subject of esthetics, and perhaps a close proximity to the divine proportions will be the best the clinician can hope for” [5]. Many proponents of the golden ratio continue to quote this paper as support for their contentions. Other well-known authorities have supported claims about the golden ratio. For example, in a comprehensive series of articles on the history of orthodontics, Dr. Norman Wahl, a modern historian of orthodontics, stated that Leonardo da Vinci had used the golden ratio [6]. Considering the widespread recognition of Leonardo as a universal genius, this in itself appears to be favourable evidence for the golden ratio. Perhaps at the more extreme end of support for the golden ratio is the following statement, from an article published in 2004 in a journal for dentists with an interest in orthodontics: “The following biologic equation holds true for all humans regardless of race, age, sex and other variables: Divine proportion = facial beauty = TMJ health = psychologic health = physiologic harmony = fertility = total health and wellness = Quality of Life” [7].

As the claims regarding the golden ratio are commonly made by orthodontists, dentists, and plastic surgeons, whether in print or at the lectern, the concept, and the assertions of its supporters, might be a subject worth exploring.

## Main text

‘One of the great commandments of science is, “Mistrust arguments from authority.”…Authorities must prove their contentions like everybody else.’ [8]

Carl Sagan (1934–96)

American astronomer, astrophysicist, and man of letters

### What is the golden ratio?

**Fig. 1:**
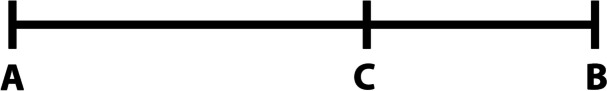
The golden ratio is a geometrical proportion in which a line AB is divided at a point C in such a way that AB/AC = AC/CB. This gives AC/AB the value 0.618… (the golden number)

The golden ratio or golden proportion is a geometrical proportion in which a line is divided at a point in such a way that the ratio of the shorter section to the longer section of the line is equal to the ratio of the longer section to the whole line (Fig. 1). This golden “ratio” forms an irrational number (1.6180339…), and an infinite decimal. The point at which the line is divided is known as the golden section, represented by the symbol Φ (Phi) derived from the name of the Greek sculptor Phidias, though this is a modern term, and there is no evidence other than unconvincing modern assertions, that Phidias employed such a proportion (see later).

### Euclid and *The Elements*

The Greek mathematician Euclid (fl. 300 BC), also known as Euclid of Alexandria and the “father of geometry”, mentions and describes such a ratio in Book II, proposition XI of his treatise *The Elements* (308 BC) [9]. At the beginning of Book VI he provides a definition (definition number 3) of what is now referred to as the golden ratio: “A straight line is said to be *cut in extreme and mean ratio*, when the whole is to one of the segments, as that segment is to the other.” Interestingly, he refers to this mode of dividing a line as “medial section”, which he says differs from “harmonical proportion”. The ratio is again mentioned briefly in Book VI, propositions XVII and XXX of his treatise, in terms of the relative proportions of rectangles formed under a line divided at such a point. However, in each of these descriptions, Euclid merely regards it as a rather general mathematical ratio. There are many propositions in each of the Books in *The Elements*, but no special mention is made of this particular proposition, and no relationship to beauty or any other aspect of the natural world is suggested. Contrary to generations of mystics and pseudoscientists who followed, Euclid soberly treats the ratio for what it is, without attaching to it any miraculous or preternatural properties. The so-called golden number itself is not mentioned [9].

### Zeising, Fechner, and Le Corbusier

**Fig. 2:**
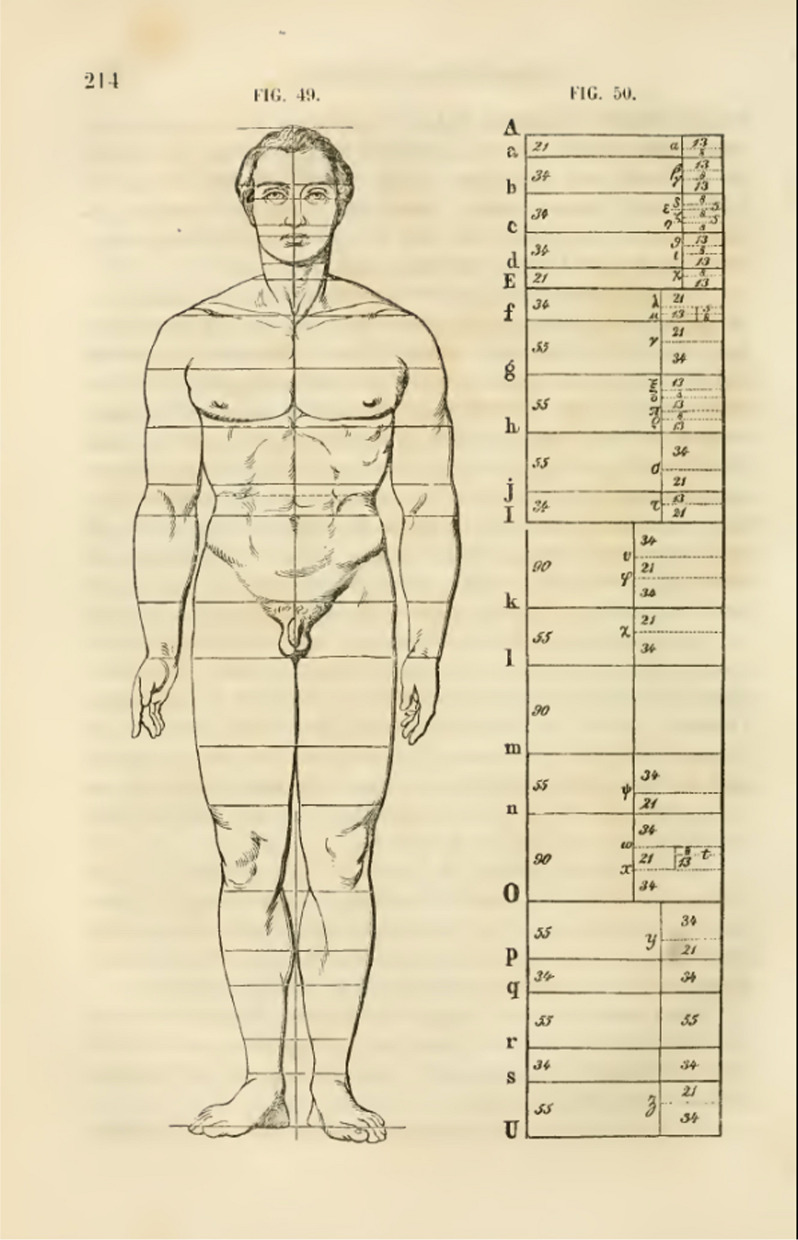
Zeising’s attempt to demonstrate the golden ratio in an “idealized male”

**Fig. 3:**
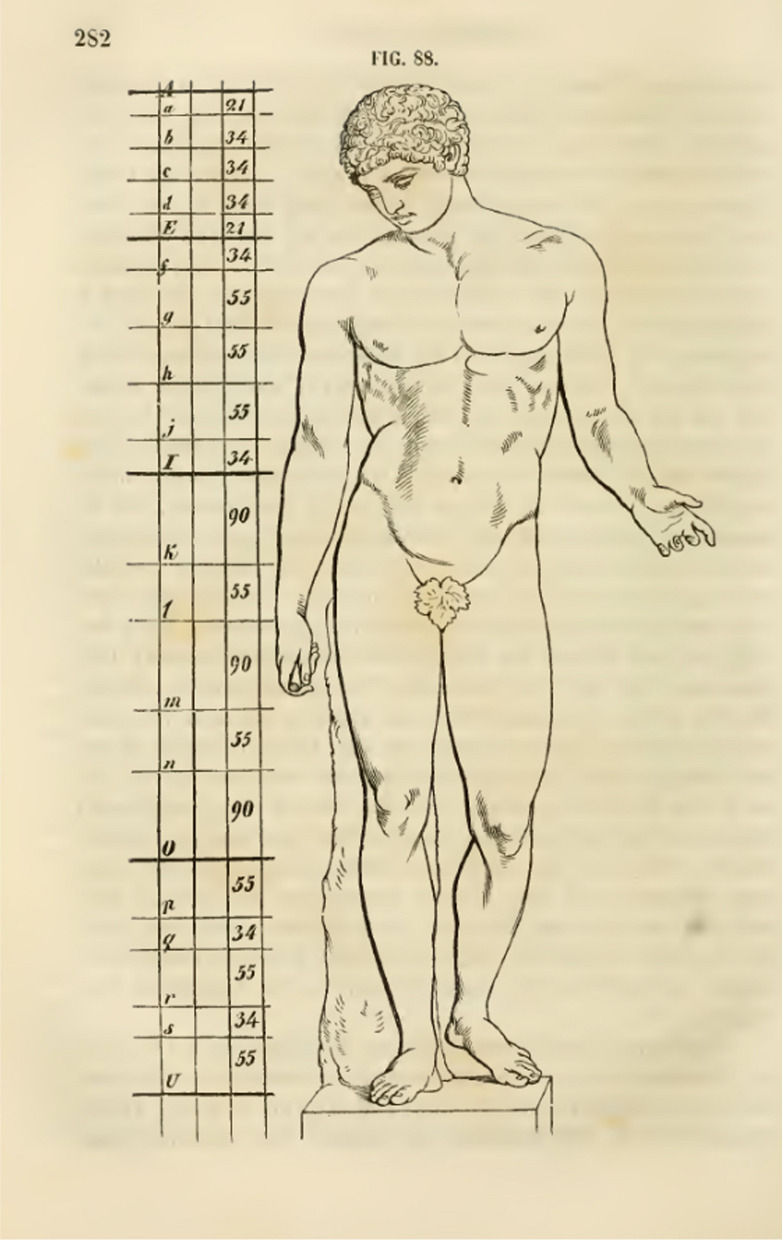
Zeising’s attempt to demonstrate the golden ratio in a Roman replica of an idealized Greek male statue

It was not until the mid-nineteenth century that the German psychologist Adolf Zeising (1810–76) tried to prove that the “golden section” was the key to morphological beauty, both in nature and in art, and particularly in the human form (Figs. 2 and 3) [10]. His description of the golden ratio as a “universal law” of beauty is revelatory of his rather overzealous views [10]:

…the universal law in which is contained the ground-principle of all formative striving for beauty and completeness in the realms of both nature and art, and which permeates, as a paramount spiritual ideal, all structures, forms and proportions, whether cosmic or individual, organic or inorganic, acoustic or optical; which finds its fullest realization, however, in the human form.

**Fig. 4:**
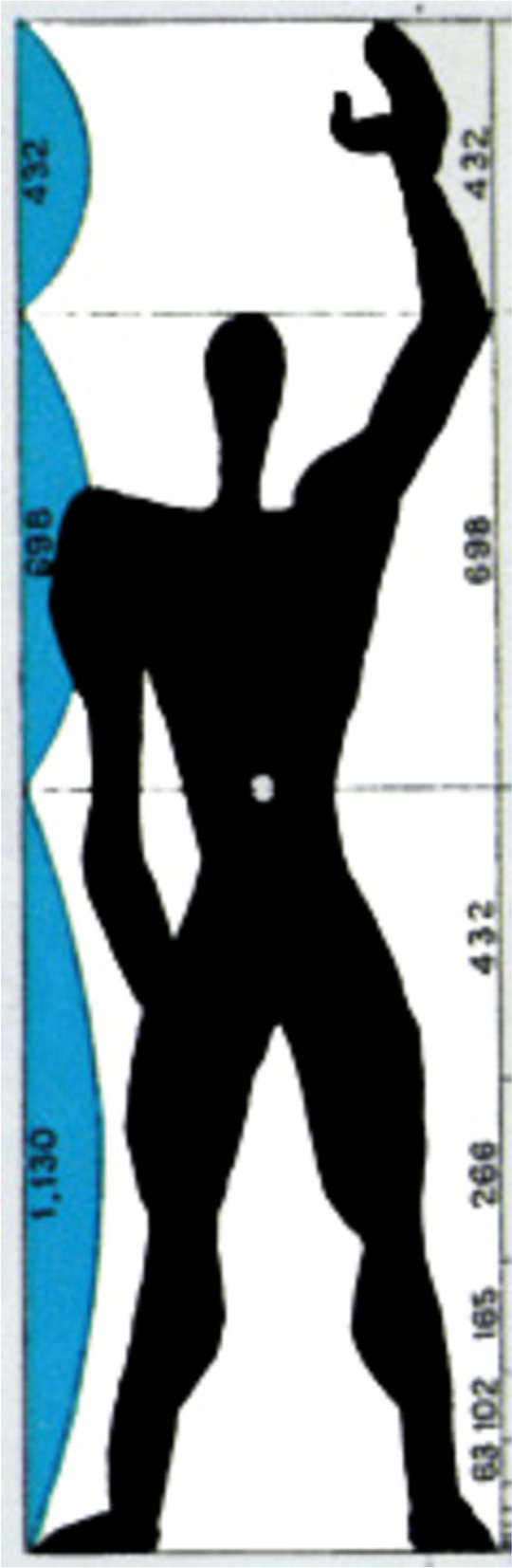
Le Corbusier’s *Le Modular* (c. 1943)

Zeising’s ideas were followed by another German psychologist, Gustav Theodor Fechner (1801–87), who constructed ten rectangles with different ratios of width to length and asked numerous observers to choose the “best” and “worst” rectangle shape, in order to compare the visual appeal of rectangles with different proportions. According to Fechner, the rectangles chosen as “best” by the largest number of participants had a ratio of 0.62 (with a range between 3:5 and 5:8) [11]. Subsequently, around 1943, another follower of Zeising, the Swiss-French architect Charles Edouard-Jeanneret, better known by his pseudonym Le Corbusier (1887–1965), described “ideal” human proportions in relation to a figure he termed *Le Modulor*, which is a rather odd looking line drawing of a human figure, which he based on the golden ratio (Fig. 4) [12]. Le Corbusier’s descriptions of *Le Modulor* and his approach to geometry demonstrate a distinctive lack of clarity, and his ideas and claims have not withstood subsequent scientific investigation in architecture [13]. Interestingly, Le Corbusier advised his architects that if a building did not look aesthetically attractive, to abandon the golden ratio and use something else [13].

### The Parthenon

**Fig. 5:**
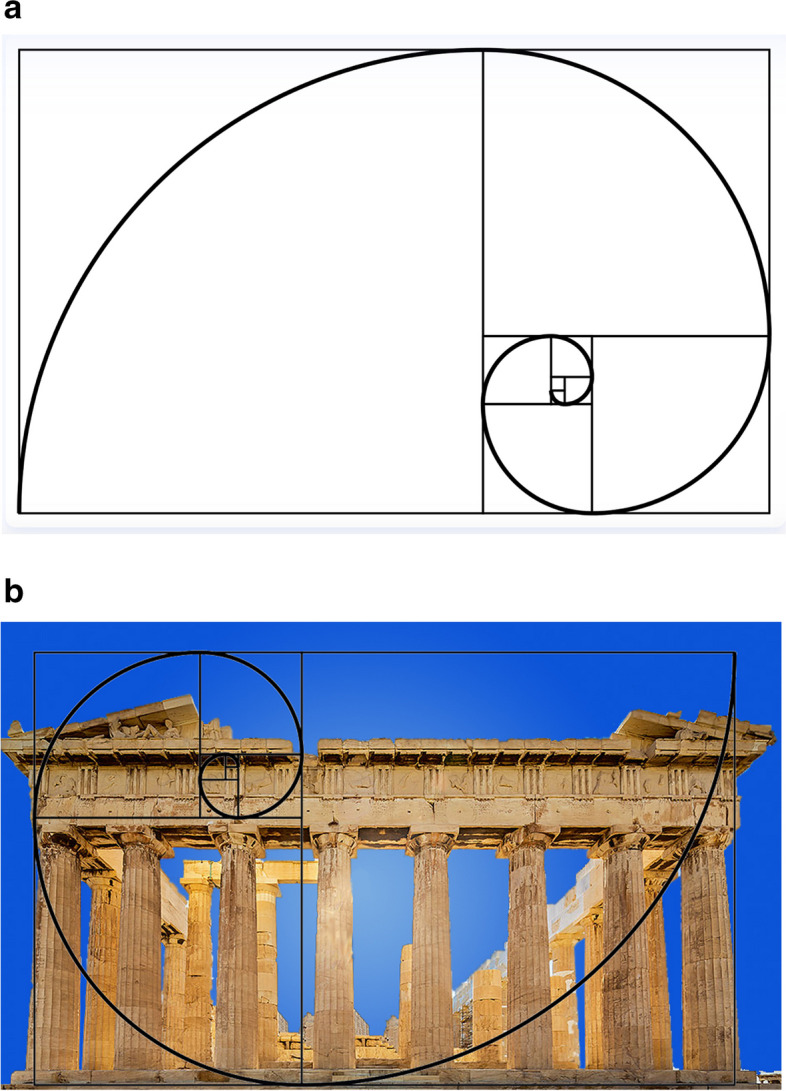
**a** The golden rectangle, containing the usual drawing of the “golden spiral”, based on the golden ratio. **b** An example of attempting to fit the golden rectangle onto the Parthenon

The Parthenon is the former temple dedicated to the goddess Athena, built in the fifth century BC on the highest part of the Athenian Acropolis. Its architect was Ictinus, assisted by another architect called Callicrates, but the building was designed and supervised by the sculptor Phidias. The often-repeated assertion that the Parthenon is based on the golden ratio is not supported by actual measurements, as mentioned above [14]. Every diagram or photograph demonstrating the “golden rectangle” (Fig. 5) on the Parthenon includes empty air above or amusingly leaves out some steps below the building. However, the entire story about the classical Greeks and the golden ratio seems to be without foundation. The same appears to be true of the pyramids of Egypt. The idea that the Parthenon and the Egyptian pyramids had been constructed according to the golden ratio dates to the mid-nineteenth century, with no mention found in any manuscript from classical times until the mid-nineteenth century. Attempts to demonstrate diagrammatically that any of these structures fit a golden rectangle or otherwise are speculative and appear to be due to extreme persistence in attempting to fit the golden ratio onto the structure. A simple internet search will demonstrate how many have attempted to place a golden rectangle onto the Parthenon. A scientific approach would involve assessing various proportions and relationships and seeing if there are any patterns, not beginning with a ratio and seeing where it will fit. This is also a disservice to mathematics and geometry, which are so important in the scientific attempts to understand the world.

### Leonardo da Vinci, Pacioli, and *De Divina Proportione*

**Fig. 6:**
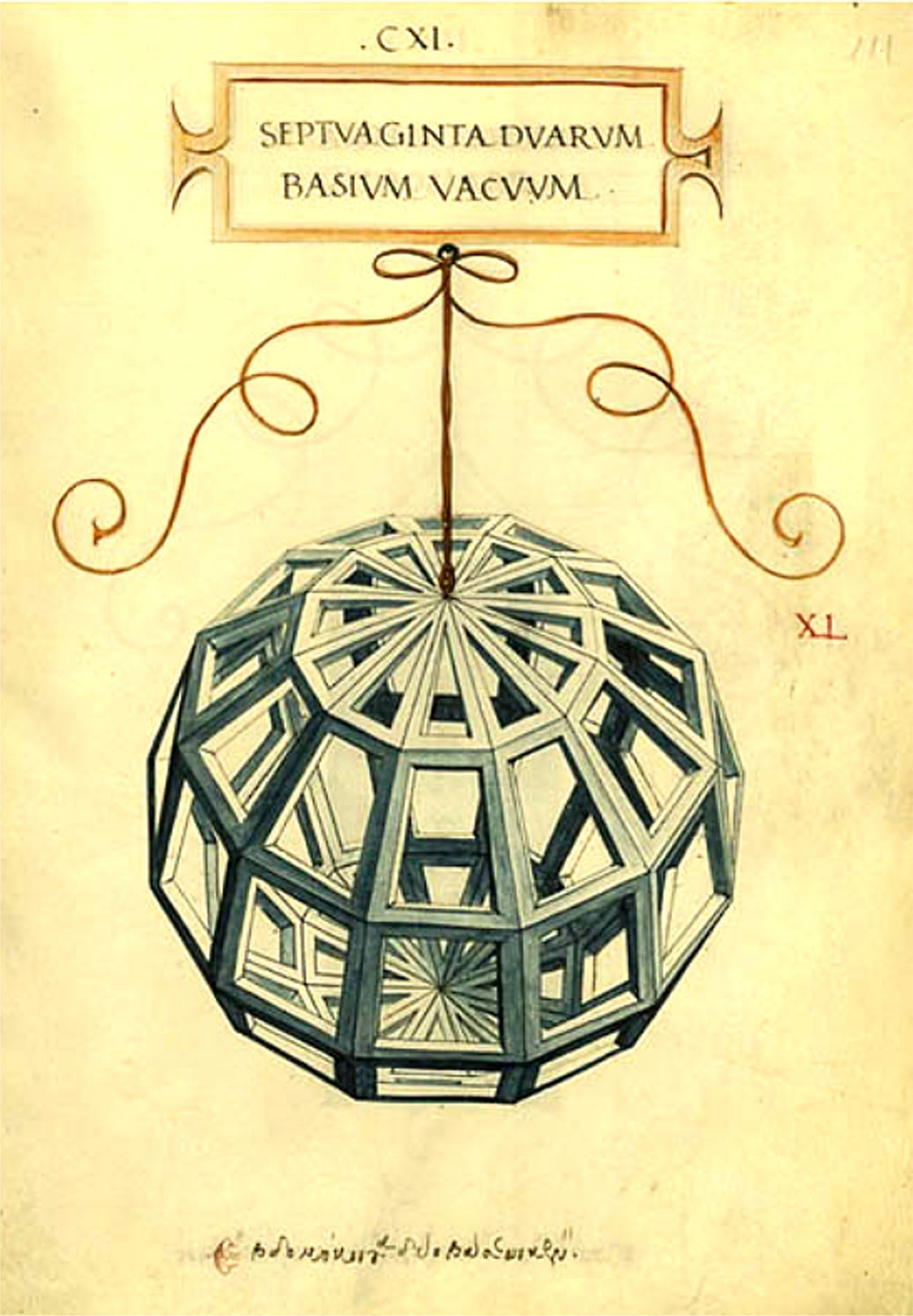
An example of a polyhedral form drawn by Leonardo da Vinci for Pacioli’s *De Divina Proportione*

Contrary to popular speculation, Leonardo da Vinci did not employ the golden ratio in any of his human-proportional drawings [15]. In 1498, the mathematician and monk Luca Pacioli (1445–1514) published his *De Divina Proportione* (On Divine Proportion). Pacioli was a religious man, demonstrated by his use of the term “divine” rather than “golden” proportion. However, contrary to popular myth, Pacioli did not argue that the golden ratio was the secret to beauty. His book is predominantly on mathematics and perspective in art. There are two simple line drawings of a facial profile in the main body of the book, the latter with the inscription “*divina proportio*”. The book also has two appendices, the first on the calligraphic drawing of capital letters, and the second with the drawings by Leonardo. In the preface, Pacioli writes that Leonardo da Vinci has drawn the geometrical figures in the second appendix of the book. It is worth noting that these are the only figures drawn by Leonardo that were published during his own lifetime. Leonardo was not a co-author (this, again, being a popular misconception), but *illustrated* the images for the second appendix of Pacioli’s book, particularly of polyhedral designs (Fig. 6). This demonstrates that Leonardo, through his acquaintance with Pacioli, must have been well aware of the concept of the golden ratio. Therefore, the fact that he does not mention the concept anywhere in his own notebooks or proportional drawings suggests that he is unlikely to have believed that the concept is related to human beauty or idealized proportions.

Of course, there will be those who will argue that Leonardo used the golden ratio in his drawings but did so secretly or as part of some conspiracy. Unfortunately, a popular novel, *The Da Vinci Code*, and a follow-up blockbuster movie made such conspiracy theories appear worthy of consideration. Bearing in mind that Leonardo’s notebooks have been studied extensively, and that we know he poured his thoughts onto their pages, the simple question arising would be why? It is up to those making such an assertion to prove their contention, and they have all their work still ahead of them.

There is another point that may at first glance appear to be a minor detail and rather pedantic, but is important. Leonardo da Vinci’s name was Leonardo. Therefore, in serious academic discussions of art and art theory, he is referred to as either Leonardo or Leonardo da Vinci, never as just “da Vinci”. The use of the latter term has increased in lay parlance, presumably due to the popularity of *The Da Vinci Code*. Therefore, when reading a paper in a surgical or otherwise scientific journal, if the author refers to Leonardo as “da Vinci”, it may be worth questioning their credibility in relation to this subject area, and their claims may need to be considered with a degree of scepticism.

The suggestion that Leonardo’s *Mona Lisa* was created employing the golden ratio is simply baseless and not supported by anything in Leonardo’s own writings. Although there has been some debate as to the identity of the sitter, it is known that the Mona Lisa is a portrait (i.e. a painting of a person), now generally accepted to be a pregnant Madonna Elisabetta, third wife of Francesco del Giocondo, not an idealized image. As such, the facial proportions are not expected to be “ideal”, even in relation to Leonardo’s own proportional canons [16].

### *Homo Vitruvianus*

**Fig. 7:**
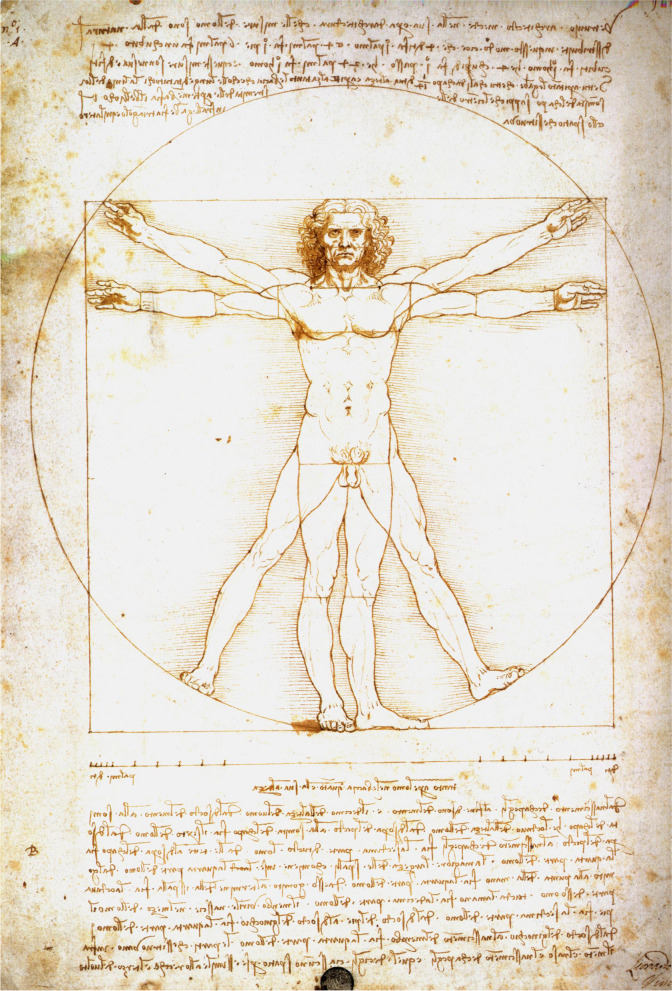
Leonardo da Vinci’s *Vitruvian Man*

What about the *Vitruvian Man*, so commonly used to support the use of the golden ratio as the basis of ideal human proportions? It is known that the classical Greeks had a profound interest in mathematics and geometry, particularly the Pythagoreans. It may be, and has been, postulated that their art and sculptures were linked in some way to their interest in mathematics, but precisely how is not known. Even the famous Canon of Polykleitos was either not recorded or was otherwise lost over time and thereby not available to the modern reader, other than through brief notes from later Roman writers and thinkers such as Pliny. However, there is a short paragraph in the work of the Roman architect Vitruvius where he compares ideal buildings to the ideal man, based on his suggestion that the proportions of the “ideal” man’s body may fit into the “perfect” geometrical shapes, the circle and square. This simple idea had a profound influence on the Renaissance mind. There were many depictions of the so-called *Homo Vitruvianus*, or *Vitruvian Man*, before and after Leonardo, but his remains are the most famous. Although the *Vitruvian Man* is often shown in connection with the golden ratio, the proportions of the figure do not actually match it, and Leonardo’s own text above and below the image only mentions whole number ratios (Fig. 7). Leonardo’s studies on human proportional relationships, intended as an aid to artists, were based on measurements of living individuals (i.e. anthropometry) [15]. Neither Leonardo nor the art theorist Leon Battista Alberti (1404–72) before him, have written anywhere that they employed the golden ratio or any other such theory in their work on human proportions [17]. Leonardo used craniofacial height as the comparative yardstick in relation to which the proportions of other body parts were measured. Interestingly, before Leonardo, Alberti had used the length of the human foot, though these two proportions are almost identical in both of their proportional canons. Side by side with Leonardo’s drawings, often on the same page, are the notes in which his mind puzzled over the laws of nature, facial beauty, and “ideal” proportions. None mention a golden ratio, proportion, or number [15].

Alberti believed that in nature there was a striving towards the ideal, which was the underlying law that regulated nature. He termed this *concinnitas*, which roughly translates as harmony through proportion. He went on to say that in the architectural design of a beautiful building or a work of art, “there resides some natural excellence and perfection that excites the mind and is immediately recognized by it” [17]. Therefore, Alberti believed in the importance of geometry and mathematics and their link to harmony, proportion, and aesthetics, yet nowhere in his works did he cite the golden ratio as the underlying nexus required for such beauty.

### The golden spiral

**Fig. 8:**
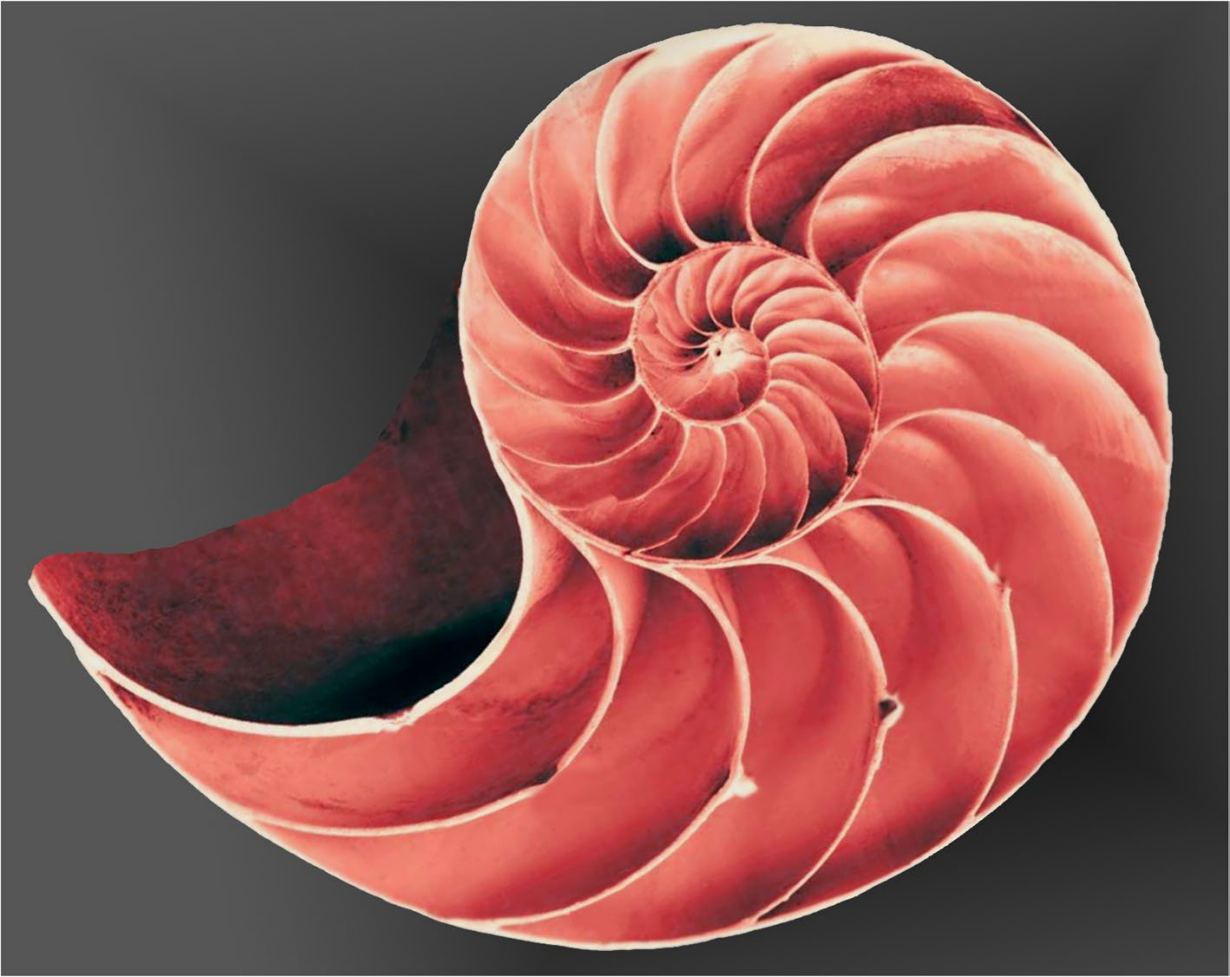
*Nautilus pompilius* shell

A “golden spiral” is supposed to get further from its central point by a factor of Φ for every quarter turn it makes, and a frequently used example to demonstrate the aesthetic qualities of the golden ratio is the *Nautilus pompilius*, a member of the octopus family (Fig. 8). However, measurements of several hundred such shells have found the average ratio to be variable, and a logarithmic spiral [18].

### The Fibonacci sequence

The Fibonacci sequence, named after the Italian mathematician Leonardo of Pisa (c. 1170–1240), is often quoted as support for the golden ratio. The Fibonacci numbers progress by adding the two previous numbers in a sequence to obtain the successive number. This number sequence was written in his book, the *Liber Abaci*, published in 1202. The *Liber Abaci* is the first European textbook of mathematics and is famous for introducing the Eastern decimal numeral system into Europe, which eventually replaced the Roman numeral system. It is a large book, with 600 pages in the English translation, but the Fibonacci sequence comprises only a small part of one rather long chapter. No specific link to beauty was described in this book, and the term Fibonacci sequence was coined by later mathematicians in the eighteenth century, as was the potential link to recurring patterns in nature and beauty. The Fibonacci sequence fractions gradually approximate but never quite meet the golden ratio. No persuasive link has been demonstrated between the Fibonacci numbers and ideal facial proportions.

### Testing the hypothesis

As with any other structure, the association between various facial proportional relationships and perceptions of attractiveness can be tested. Admirers and supporters of the golden ratio begin with the ratio in mind, then attempt to prove its existence in the attractive human face. The choice of landmarks is often arbitrary, as is head positioning in photographs, making the genuine test and subsequent confirmation or refutation of the theory appear unverifiable. Those who begin by looking for confirmations of their desired theory will find confirmations somewhere. The style of writing from such authorities, even in otherwise scientific journals, suggests that the golden ratio will be found in any beautiful structure in nature, including the human face, and that this is self-evident to those who can see it, but hidden from the uninitiated. The suggestion is that once the unbeliever’s eyes have been figuratively opened, they will find verifications everywhere. The literature is replete with articles on the golden ratio/proportion, as a simple search of the main electronic databases will demonstrate. However, most scientific investigations have found no plausible relationship between improved facial appearance and the golden ratio, whether in orthodontic or orthognathic patients [19, 20], or the faces of professional models from different ethnic backgrounds [21, 22]. There are many publications on the golden ratio in the literature, but these few appear to have unbiased methodology. The idea that the golden ratio should be used as an aim in dentofacial treatment, whether orthodontic or surgical or as an aim to improve attractiveness, appears to have dubious validity.

### The perpetuation of myths

No serious book on art or mathematics makes any such claims about the mysterious properties of the golden ratio in relation to human proportions or aesthetics. This begs the perplexing question of why so many otherwise serious clinical books, particularly in dentistry and plastic surgery, continue to promote this concept. The potential reason appears twofold, albeit speculative and perhaps uncomfortable to discuss. Firstly, the authors of such works may believe that no one in their readership will have read, or is ever likely to read, the original works of Euclid, Pacioli, or Leonardo. Moreover, the authors themselves may not have read the original works, but simply re-quote from previous publications, though always citing the original works, which leads to a perpetuation of myths. The American scientist Stephen Jay Gould (1941–2002) wrote [23]: “very few people, including authors willing to commit to paper, ever really read primary sources—certainly not in necessary depth and contemplation, and often not at all.”

Secondly, there may be a lack of application of the scientific method, with its central tenets that science is not a system of beliefs, that assertions require justifiable evidence, and that the scientific method demands replication of results. Each phenomenon must be re-examined, preferably by independent and impartial investigation, and the interpretation given to it confirmed or discarded through dispassionate analysis and experimentation [24]. As Karl Popper (1902–94), the leading philosopher of science explained, the re-examination must be critical [25]:

If we are uncritical we shall always find what we want: we shall look for, and find, confirmations, and we shall look away from, and not see, whatever might be dangerous to our pet theories. In this way it is only too easy to obtain what appears to be overwhelming evidence in favour of a theory which, if approached critically, would have been refuted.

### Factors contributing to the perpetuation of myths

Many beliefs about the golden ratio in aesthetics appear to be based on misconceptions or exaggerations. It is interesting to analyse the psychological underpinnings and sociocultural factors contributing to the perpetuation of the golden ratio myth in aesthetics. Exploration of why this myth persists despite the lack of empirical evidence may provide valuable insights into human tendencies to find patterns and meanings in nature and art.

The scientific mindset requires an unbiased capacity for the evaluation of uncertain or conflicting information. There are two types of bias to which humans are not immune: conviction bias and confirmation bias. The former occurs when we desperately want something to be true, and thereby mentally convince ourselves of its ‘truth’. The latter occurs when we consistently search for evidence to support our preferred view. For example, we may read a scientific article’s abstract or conclusions that support our preferred theory, rather than reading and scrutinizing the methodology and results, which may shed light on shortcomings in the research methods that would make the conclusions unsustainable.

Although humans are not immune from these forms of bias, the scientific method, at its best, should be immune to them, and is said to be ‘self-correcting’, i.e. our understanding improves and changes as new information and evidence becomes available. However, there are potential human-induced impediments to the self-correction process, which may be responsible for the perpetuation of myths and the maintenance of unchallenged fallacies. Ioannidis [26, 27] has suggested that self-correction in science does not occur by default. Most research papers are not replicated, predominantly because most journals are not interested in replication studies [28, 29], and often not interested in the publication of negative results [30]. Alternatively, if the replication method in an investigation contains the same methodological or underlying assumption errors as the original investigation, the results can lead to a perpetuation fallacy [31, 32]. As such, it is possible for perpetuated and unchallenged fallacies to comprise the majority of the circulating evidence in a specific field [33]. Although replication evidence is uncommon, when it does occur, it is usually not by independent and impartial investigators, but by the same investigators, or their followers, and, unsurprisingly, proposing the same original findings [26].

Potential areas for further investigation do exist. The possibility of links between mathematics, geometry, and dentofacial aesthetics continues to require exploration. However, researchers should approach their work without unshakeable pre-existing convictions. The methodology should be sound and the analysis of results impartial. Moreover, the validity of ideas expressed by others based on the ‘evidence’ they have supplied does not have to be accepted at face value but can be examined sceptically, and that includes any information provided in this article.

When assessing an article, whether evaluating a ratio in human faces or other objects, whether natural or man-made, the question we should ask is are the landmarks used by the investigators arbitrary, and designed to fit a specific ratio? If so, why have they been chosen? It would be better to assess valid dentofacial proportions, or the proportions of any object under investigation (be it the Parthenon or the Mona Lisa), based on logical landmarks and interlandmark measurements, and assess the ratios found, whatever they may be, in order to see if any patterns exist.

## Conclusions

The mathematical and geometrical relationships between facial beauty, proportions, and perceptions of attractiveness require further investigation. There is currently no convincing evidence to support the use of the golden ratio in orthognathic or facial aesthetic/reconstructive surgical planning or the analysis of treatment results.

The golden ratio does not seem to appear in most of the contexts with which it is cited to be related, and many of the claims regarding it are unreliable. Our understanding of exactly what makes a face beautiful still requires investigation. Unfortunately, most of the “evidence” put forward to support the golden ratio as the secret to beauty appears to be without foundation. This demonstrates how myths and misconceptions can be perpetuated in science unless all assertions are approached with a level of scientific scrutiny. Great conviction in often deeply held beliefs, no matter how psychologically attractive, is not a substitute for evidence. “Convictions”, wrote Nietzsche, “are more dangerous enemies of truth than lies” [34].

## Data Availability

Not applicable.
